# Behavioral Addictions Prevalence and Impact on Medical Sciences Students' Mental Health: A Systematic Review and Meta-Analysis

**DOI:** 10.1192/bjo.2024.168

**Published:** 2024-08-01

**Authors:** Omer A. Mohammed, Danya Ibrahim, Shima Algam Mohamed, Shaza K. Babikir, Ayman Zuhair

**Affiliations:** 1 Khartoum University, Faculty of Medicine, Khartoum, Sudan; 2 University of Al-Neelain, Faculty of Medicine, Khartoum, Sudan; 3 Norfolk and Suffolk NHS Foundation Trust, Norwich, United Kingdom

## Abstract

**Aims:**

This study aims to illuminate the prevalence of various behavioral addictions among health professions students and examine their negative effects on mental health.

**Methods:**

In March 2023, a systematic literature search was conducted, encompassing randomized controlled trials, cohort, case-control, and cross-sectional studies from the past five years in PubMed and ScienceDirect. Adhering to the Preferred Reporting Items for Systematic Reviews and Meta-Analyses (PRISMA) guidelines, 19 papers underwent qualitative analysis, while 15 studies were subjected to quantitative analysis following a quality assessment review.

**Results:**

The study included 9,994 health professions students, primarily in the field of medicine, aged between 18 and 23 years. The majority of the students were unmarried, females, and most were in clinical years. The prevalence of behavioral addiction was 36% (95% CI: 20–51), with smartphone addiction being the highest at 46%, followed by internet addiction (42%), social media disorder (22%), and gaming disorder (4%). Substantial heterogeneity was observed among the studies. A funnel plot analysis assessed the potential for publication bias, revealing no significant indication of bias. A significant difference was observed between the groups.

**Conclusion:**

This study identifies five distinct forms of behavioral addictions influencing the mental health and daily activities of health professions students. The findings underscore the need for longitudinal and interventional studies to address this technological threat.

